# Differences in Incidence and Risks of Suicide Attempt and Suicidal Drug Overdose between Patients with Epilepsy with and without Comorbid Depression

**DOI:** 10.3390/ijerph16224533

**Published:** 2019-11-15

**Authors:** Chi-Yu Lin, Tomor Harnod, Cheng-Li Lin, Wei-Chih Shen, Chia-Hung Kao

**Affiliations:** 1 Stroke Care Center and Department of Neurology, Yumin Hospital, Nantou 542, Taiwan; justherblin@gmail.com; 2 Department of Neurosurgery, Hualien Tzu Chi Hospital, Buddhist Tzu Chi Medical Foundation, Hualien 970, Taiwan; tomorha@yahoo.com.tw; 3 College of Medicine, Tzu Chi University, Hualien 97004, Taiwan; 4 Management Office for Health Data, China Medical University Hospital, Taichung 404472, Taiwan; orangechengli@gmail.com; 5 College of Medicine, China Medical University, Taichung 40402, Taiwan; 6 Department of Computer Science and Information Engineering, Asia University, Taichung 41354, Taiwan; wcshen@gmail.com; 7 Center of Augmented Intelligence in Healthcare, China Medical University Hospital, Taichung 404472, Taiwan; 8 Graduate Institute of Biomedical Sciences and School of Medicine, College of Medicine, China Medical University, Taichung 40402, Taiwan; 9 Department of Nuclear Medicine and PET Center, China Medical University Hospital, Taichung 404472, Taiwan; 10 Department of Bioinformatics and Medical Engineering, Asia University, Taichung 41354, Taiwan

**Keywords:** cohort study, depression, epilepsy, suicide, National Health Insurance

## Abstract

*Objective*: To determine the differences in the incidences and risks of suicide attempt (SA) and suicidal drug overdose (SDO) between patients with epilepsy with and without comorbid depression by using data from Taiwan’s National Health Insurance Research Database. *Methods*: We analyzed data of patients (≥20 years) who had received epilepsy diagnoses between 2000 and 2012; the diagnosis date of epilepsy was defined as the index date. The epilepsy patients were divided into the cohorts, with and without comorbid depression, and compared against a cohort from the non-affected population. We calculated adjusted hazard ratios and the corresponding 95% confidence intervals for SA and SDO in the three cohorts after adjustment for age, sex, and comorbidities. *Results*: The incidences of SA and SDO in the cohort with epilepsy and depression were 42.9 and 97.4 per 10,000 person-years, respectively. The epilepsy with depression cohort had 21.3 times of SA risk; and 22.9 times of SDO risk than did the comparison cohort had a 6.03-fold increased risk of SA and a 2.56-fold increased risk of SDO than did the epilepsy patients without depression. Moreover, patients’ age <65 years, and female sex would further increase the risk of SA in patients with epilepsy and comorbid depression. *Conclusion*: Risks of SA and SDO in patients with epilepsy are proportionally increased when depression is coexisted. Our findings provide crucial information for clinicians and the government for suicide prevention and to question whether prescribing a large number of medications to patients with epilepsy and depression is safe.

## 1. Introduction

Both epilepsy and depression are common brain disorders worldwide. The patients with depression may experience low self-confidence, have feelings of guilt or self-blame, and have more suicidal ideation and suicidal behaviors than do healthy individuals [1]. Most suicidal behaviors are observed in patients with psychiatric disorders, and depression is the most critical and popular one [2]. In our mind, improving the effectiveness of depression treatment and interrupting the suicidal behaviors are central to suicide prevention. Moreover, depression could be an idiopathic disease or a comorbid disorder coexisting with another brain disorder [3]. Among them, a high prevalence of comorbid depression was estimated in patients with epilepsy [4].

Epilepsy has a clinical prevalence of 0.5–1% in the general population [5,6], although it is considered with a higher prevalence by electro-encephalography. Based on its etiology, epilepsy can be categorized into idiopathic epilepsy and structural epilepsy, which are comorbid with various types of brain diseases [7]. Epilepsy not only affects the quality of life of patients, but also causes premature death [8,9]. Among all etiologies of premature death in patients with epilepsy, suicidal death is estimated to account for 5% of deaths in such patients [8,9,10]. Most studies on suicidality with epilepsy have been conducted in developed Western countries; therefore, the data reported in these studies may not be applicable to populations in underdeveloped or developing Asian countries because of the differences in the ethnic and cultural backgrounds between Asian and Western societies [11,12]. Such differences might affect the risk of suicide attempt (SA) in a patient before the suicide is completed. In addition, several confounding effects of various psychiatric or mental comorbidities may be observed in patients with epilepsy before they attempt suicide. We know that suicidality exists in psychiatric diseases independently. Previous studies have tried to explain the link between depression, epilepsy, and suicidality. The reported mechanisms seemed to be different between those of depression alone and SA, and those of epilepsy alone and SA. There were little evidence regarding the different risks of SA between patients with epilepsy with or without comorbid depression [13]. We believe that improving the understanding and reducing the risk of SA in patients with epilepsy with comorbid depression is vital for effective prevention of SA in these patients.

The Taiwanese government has established a health care system covering approximately 99% of the residents for the past two decades [14]. In this study, we used the National Health Insurance Research Database (NHIRD) of Taiwan to determine the differences in the risk of SA between patients with epilepsy with and without comorbid depression. Because Taiwan is located in East Asia and Taiwanese society is similar to Chinese and Southeast Asian societies [12], our findings might help in the development of an efficient prevention system for SA in Taiwan and other East Asian countries, which can, thus, reduce the care burden on patients’ families and the government.

## 2. Methods

### 2.1. Data Source

Data from the NHIRD of Taiwan are released by the National Health Insurance Administration and contain detailed health and medical treatment information of insurants, such as inpatient, outpatient, medication, operation treatment, etc. In this study, we used the inpatient file of the whole population to explore the association between the presence of depression in patients with epilepsy and SA incidence. The Ethics Review Board of China Medical University (CMUH104-REC2-115-CR4) approved this study. In addition, all history diagnoses in the database were coded according to the International Classification of Diseases, Ninth Revision, Clinical Modification (ICD-9-CM).

### 2.2. Study Population

The epilepsy cohort comprised patients who were aged ≥20 years and had received a new diagnosis of epilepsy (ICD-9-CM codes 345) between 2000 and 2012; the diagnosis date of epilepsy was defined as the index date. According to the status of depression diagnosis (ICD-9-CM codes 296.2, 296.3, 296.82, 300.4, and 311), the epilepsy cohort was divided into two sub-cohorts: Epilepsy without depression (EPN) and epilepsy with comorbid depression (EPD). We excluded patients aged <20 years and those with a record of SA (ICD-9-CM codes E950-E959) before the index date. The comparison cohort was selected using the same exclusion criteria, and the individuals in the cohort were matched according to age (5 years), sex, and index year at a ratio of 1:2; the individuals in the cohort were randomly assigned an index date. To obtain a deep understanding of the effect of long-term prescription on the risk of a specific type of SA- suicidal drug overdose (SDO) in the EPD cohort, the codes of SDO (ICD-9 CM codes 960–979, but without E codes) and SA (ICD-9-CM codes E950-E959) were individually analyzed in our patients with epilepsy. In total, 54,520 patients with epilepsy and 109,040 matched controls were followed up until the occurrence of SA or SDO, death, or the end of the study on 31 December 2013, whichever occurred first. Mental-health-associated diseases that were diagnosed before the index date were defined as comorbidities and included schizophrenia (ICD-9-CM code 295), alcohol-related illness (ICD-9-CM codes 291, 303, 305.00, 305.01, 305.02, 305.03, 571.0, 571.1, 571.3, 790.3, and V11.3), anxiety (ICD-9-CM code 300.00), mental disorders (ICD-9-CM codes 290–319), and insomnia (ICD-9-CM codes 307.4 and 780.5).

### 2.3. Statistical Analysis

The distributions of sex, age, monthly income, urbanization level, occupation category, and comorbidity are described as numbers and percentages in the total epilepsy, EPN, EPD, and comparison cohorts. We stratified the patients with their age, as old aged (≥65 years old), middle aged (50–64 years old), and younger (20–49 years old) subgroups for further age-related analysis. The differences between the total epilepsy cohort and comparison cohort were tested using the chi-square test and *t*-test. Hazard ratios (HRs) and 95% confidence intervals (CIs) were estimated using Cox proportional hazard models for evaluating the association between epilepsy, epilepsy with comorbid depression, and potential risk factors and SA and SDO. A multivariate Cox proportional hazard model was used to estimate adjusted HRs (aHRs) after adjustment for age; monthly income; urbanization level; and comorbidities of schizophrenia, depression, alcohol-related illness, anxiety, mental disorders, and insomnia. Analyses after stratification by age, sex, and comorbidity were performed for determining the association between epilepsy with and without comorbid depression and SA in different populations. In addition, the significance of interactions between different variables and epilepsy, as well as interactions between different variables and the incidence of depression among patients with comorbid epilepsy was evaluated. Cumulative incidence curves of SA and SDO were computed using the Kaplan–Meier method, and the differences in the curves of the comparison, EPN, and EPD cohorts were tested using a log-rank test. All statistical analyses were performed using SAS statistical software, Version 9.4 (SAS Institute Inc., Cary, NC, USA). Kaplan–Meier curves were plotted using R software. Statistical significance was determined using 2-tailed tests (*p* < 0.05).

## 3. Results

Of the individuals constituting the overall study cohort, 62.6% were men, and 37.4% were women. The mean (standard deviation [SD]) age was 53.8 (19.9) years in the total epilepsy (EPD+EPN) cohort and 53.5 (19.9) years in the comparison cohorts. The total epilepsy cohort and comparison cohort were significantly different with respect to the distributions of monthly income, urbanization level, and comorbidities. Moreover, the total epilepsy cohort had significantly higher proportions of patients with schizophrenia, alcohol-related illness, anxiety, mental disorders, and insomnia than did the comparison cohort (Table 1).

**Figure 1:**
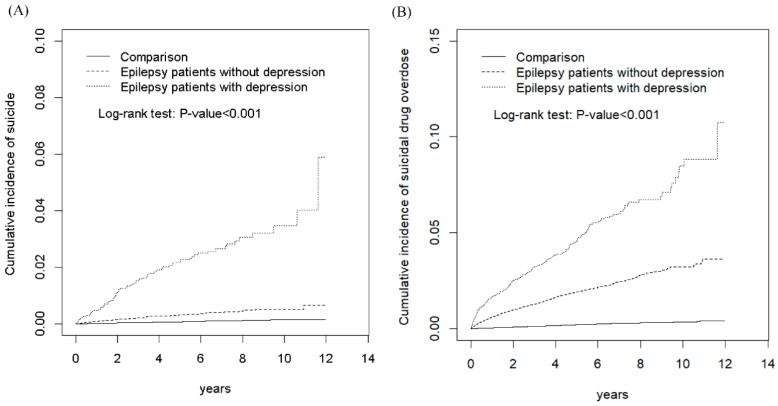
Comparison of cumulative incidence of suicide attempt (**A**) and suicidal drug overdose (**B**) among patients with epilepsy with comorbid depression, patients with epilepsy without comorbid depression, and comparison individuals from the non-affeced population.

Figure 1 presents the cumulative incidence curves of SA and SDO in the EPD, EPN, and comparison cohorts. The results revealed significant differences in the cumulative incidence curves of SA between the EPD, EPN, and comparison cohorts, with the *p*-value for the log-rank test being <0.001 (Figure 1A). Moreover, the results showed significant differences in the cumulative incidence curves of SDO between the EPD, EPN, and comparison cohorts, with the *p*-value for the log-rank test being <0.001 (Figure 1B). Table 2 shows the incidence, HRs, and 95% CIs of various risk factors for SA. The incidences of SA in the comparison, total epilepsy, EPN, and EPD cohorts were 1.46, 10.5, 6.36, and 42.9 per 10,000 person-years, respectively. Compared with the comparison cohort, the total epilepsy, EPN, and EPD cohorts exhibited significantly higher aHRs (95% CIs) for SA (5.49 [4.13, 7.29], 3.48 [2.54, 4.77], and 21.3 [15.2, 30.0], respectively). In addition, patients with relatively low monthly income, with alcohol-related illness, without mental disorders, and with insomnia exhibited significantly higher risks of SA when compared with other patients (Table 2).

Table 3 shows the results of the stratified analysis performed for the association between epilepsy and the risk of SA. Among patients aged <65 years, those in the total epilepsy, EPN, and EPD cohorts had significantly higher aHRs for SA than did those in the comparison cohort. Patients aged <65 years in the EPD cohort had an extremely higher risk of SA than did the individuals in the comparison cohort; however, the risk of SA in patients aged ≥65 years in the total epilepsy cohort did not differ significantly from that in the individuals in the comparison cohort. The results showed a significant interaction between age and epilepsy (*p* < 0.001) and between age and depression (*p* < 0.001) among the patients with epilepsy. When the sex of the patients was considered for analysis, women in the total epilepsy, EPN, and EPD cohorts exhibited significantly higher aHRs (95% CIs) for SA (8.49 [5.23, 13.8], 4.53 [2.64, 7.77], and 35.5 [20.8, 60.5], respectively) compared with those in the comparison cohort. Men in the total epilepsy, EPN, and EPD cohorts had significantly higher aHRs (95% CIs) for SA, which were 4.15 (2.90, 5.93), 2.93 (1.98, 4.33), and 13.8 (8.64, 22.1), respectively, compared with those in the comparison cohort. A significant interaction was noted between sex and epilepsy incidence (*p* = 0.05) and between sex and depression among patients with epilepsy (*p* = 0.004). Among the subgroups of patients with different monthly income levels, the total epilepsy, EPN, and EPD cohorts exhibited significantly higher aHRs for SA compared with the comparison cohort, and significant interactions between monthly income and epilepsy (*p* = 0.02) and between monthly income and depression were observed among patients with epilepsy (*p* = 0.02). Among the subgroups of the patients living in regions with different urbanization levels and different occupations, the total epilepsy, EPN, and EPD cohorts had significantly higher aHRs for SA compared with the comparison cohort. Moreover, urbanization level and occupation significantly interacted with depression among patients with epilepsy. Among patients without comorbidities, the total epilepsy, EPN, and EPD cohorts exhibited significantly higher aHRs (95% CI) for SA (5.25 [3.89, 7.09], 3.41 [2.43, 4.77], and 24.7 [17.1, 35.7], respectively) compared with the comparison cohort. Among patients with one or more comorbidities, the total epilepsy and EPD cohorts, but not the EPN cohort, had significantly higher aHRs (95% CIs) for SA (2.76 [1.11, 6.87] and 9.14 [3.58, 23.3], respectively) compared with the comparison cohort (Table 3).

Because we determined that depression exhibited a significantly positive association with SA in patients with epilepsy, we further compared the EPD cohort to the EPN cohort (Table 4). The EPD cohort had a significantly higher aHR (95% CI) for SA (6.03 [4.40, 8.28]) than did the EPN cohort. Moreover, among patients aged <65 years, the EPD cohort had a significantly higher risk of SA than did the EPN cohort. A significant interaction was observed between depression and age (*p* = 0.03). Both women and men in the EPD cohort exhibited high risks of SA, with the corresponding aHRs (95% CI) being 7.98 (5.05, 12.6) and 4.49 (2.86, 7.04), respectively. Among the subgroups of patients with epilepsy with different monthly income levels, living in areas with different urbanization levels, and with different occupation categories, the EPD cohort had significantly higher aHRs for SA compared with the EPN cohort. Irrespective of the presence of any comorbidity in patients with epilepsy, the EPD cohort had significantly higher aHRs (95% CIs) for SA (7.50 [5.20, 10.8] and 5.64 [3.77, 8.44] in patients without and with comorbidities, respectively) than did the EPN cohort (Table 4).

Epilepsy and depression were determined to be significantly positively associated with SDO after adjustment for age; monthly income; urbanization level; and comorbidities of schizophrenia, alcohol-related illnesses, anxiety, mental disorders, and insomnia. The incidences of SDO in the comparison, total epilepsy, EPN, and EPD cohorts were 3.88, 45.8, 39.3, and 97.4 per 10,000 person-years, respectively. Compared with the comparison cohort, the total epilepsy, EPN, and EPD cohorts had significantly higher aHRs (95% CI) for SDO (10.6 [8.99, 12.4], 9.01 [7.63, 10.6], and 22.9 [18.7, 28.2] for SDO, respectively). Compared with the EPN cohort, the EPD cohort had a significantly higher aHR (95% CI) for SDO, which was 2.56 (2.17, 3.04), as presented in Table 5. Moreover, the EPD cohort had higher aHRs (95% CI) for poisoning by medicinal substances, tranquilizers, and others (3.67 [2.14, 6.32], 6.24 [3.72, 10.5], and 2.19 [1.80, 2.65], respectively) than did the EPN cohort.

## 4. Discussion

The present retrospective study involved a 13-year follow-up of patients aged ≥20 years, and the results reveal that 10.35% (5641 out of 54,520) of Taiwanese patients with epilepsy would have comorbid depression. The incidences of SA in the comparison, EPN, and EPD, and cohorts were 1.46, 6.36, and 42.9 per 10,000 person-years, respectively, and patients in the EPD cohort had a strongly increased (21.3 times) risk of SA. By contrast, patients without EPD had a moderately high (3.48 times) risk of SA, and the data are within the range reported in a previous study in the United Kingdom [15]. According to our findings, the prevalence of comorbid depression in Taiwanese patients with epilepsy was lower than a previously reported range of 30–35% [4]. Depression should be considered a major comorbid disorder that is associated with another brain disorder and increases the risk of SA. However, depression is usually underdiagnosed and undertreated in many Western countries, including Germany [16]. Wittchen et al. reported that only one-third of individuals who met the diagnostic criteria for depression received a diagnosis of depression and that less than one-third of diagnosed patients received appropriate treatment. A study in Canada revealed that only 14% of patients with depression received a diagnosis of depression and that 85% of them received appropriate treatment [17]. These results demonstrate that only 9% to 12% of patients with depression receive adequate treatment in developed Western countries.

SA involves violent and nonviolent methods. Two recent studies conducted on poststroke patients have revealed that the leading method of SA in patients with a physical disability was self-poisoning [18,19]. In Asian countries, patients usually attempt suicide by using nonviolent methods, such as self-poisoning, which may be easier to perform than violent methods [20,21]. However, SA by self-poisoning still exerts a considerable burden on various medical care systems and governments in Asian countries. This study complementarily revealed that depression proportionally increased the risks of SA and SDO in patients with epilepsy. The incidences of SDO through drugs obtained from Taiwan’s medical care system were 3.88, 39.3, and 97.4 per 10,000 person-years respectively in the comparison, EPN, and EPD cohorts, with the aHR of SDO in the EPD cohort being as high as 22.9. Moreover, our results show that self-poisoning with medical substances or tranquilizers is the most common nonviolent method of SA in patients with epilepsy in developing Asian countries, such as Taiwan [21]. Once a patient with depression and another brain disorder starts using a large number of medications, the patient obtains access to the easiest method of attempting suicide is taking all medications at once. The results could potentially have an impact on public policy when considering that medical substances or tranquilizers obtained from the medical care system can be abused by patients with epilepsy and comorbid depression for attempting suicide. Accordingly, caution should be exercised in the provision of a long-term prescription with large amounts of medications in Taiwan, particularly to patients who have received the diagnosis of epilepsy with comorbid depression.

Many factors should be considered during the development of a suicide prevention system: Such factors include differences in cultural, ethnic, and socioeconomic backgrounds; availability of firearms; age; chronic disorders in society; financial status of individuals and families; and personal philosophy for facing job loss, divorce, and other negative life experiences. The differences between the results obtained in Taiwan and those obtained in other countries might be crucial for designing future SA prevention systems in Taiwan and other Asian countries. However, the detailed mechanisms and interactions between epilepsy, depression, and SA or SDO are currently under investigation. First, patients with epilepsy are considered to exhibit increased suicidality. Epileptogenesis increases neuroinflammation, which may result in abnormalities in the blood-brain barrier function, glutamate regulation, microglia activation, and pathological autoimmune responses. All the aforementioned conditions facilitate the development of neuropsychiatric disorders, such as depression or mental disorders, and then result in SA and SDO [22,23]. In addition, some bodies of evidence suggest that the use of antiepileptic drugs might adversely affect the mood of patients with epilepsy, and result in the development of depression [24,25]. The present study demonstrated that patients in the EPD cohort specifically had a 6.03 times higher risk of SA and 2.56 times higher risk of SDO than did those in the EPN cohort.

Our large-scale study, which was moderately unselective, suggests that the coexistence of depression and epilepsy should be considered a serious warning of SA and SDO. Our findings would not only be valuable for clinicians and the government of Taiwan, but also provide a reference for other Asian countries whose heritage is similar to that of Taiwan. However, this study has several limitations. First, we identified SA, SDO, epilepsy, and depression on the basis of the ICD-9-CM coding system; patients with minor SA or SDO may not have sought medical service, and thus, some underestimation of SA and SDO is possible. Nevertheless, the National Health Insurance Administration performs regular reviews to ensure that the files are accurate, and any false claim would be heavily penalized; therefore, miscoding might rarely occur in the NHIRD. Second, we could not directly contact the patients because their identities are anonymized in the NHIRD. Therefore, details regarding personal psychosocial factors, such as individual’s suicidal ideation, severity scores of personal stress, the severity of epilepsy, or how their epilepsy and depression were treated in the cohorts, could not be further analyzed. The actual uses of antiepileptic drugs, antidepressant drugs, or psychotherapy in patients are confounding factors associated with their risks of SA and SDO. Third, although our study design included adequate controls for numerous confounding factors, unmeasured or unknown confounders may exist as bias. However, despite the aforementioned limitations, this nationwide population-based study adequately demonstrated a significant relationship between SA and SDO and depression in patients with epilepsy.

## 5. Conclusions

The risks of SA and SDO in Taiwanese patients are increased by epilepsy and comorbid depression. Moreover, patients’ age <65 years and female sex would further increase the risk of SA in patients with epilepsy and depression. Our findings provide crucial information for clinicians and the government for suicide prevention and to question the safety of prescribing a large number of medications to patients with epilepsy and depression. Further detailed studies in future are necessary to determine whether those are applicable globally.

## Figures and Tables

**Table 1:** Distributions of age, sex, and comorbidities between epilepsy and comparison cohorts.

Variable	Total Epilepsy N = 54,520		Epilepsy without Depression N = 48,879		Epilepsy with Depression N = 5641		Comparison Cohort N = 109,040		*p*-Value
	*n*	%	*n*	%	*n*	%	*n*	%	
Sex									0.99
Women	20,368	(37.4)	18,044	(36.9)	2324	(41.2)	40,736	(37.4)	
Men	34,152	(62.6)	30,835	(63.1)	3317	(58.8)	68,304	(62.6)	
Age stratified									0.99
≤49	24,512	(45.0)	21,724	(44.4)	2788	(49.4)	49,024	(45.0)	
50–64	11,703	(21.5)	10,508	(21.5)	1195	(21.2)	23,406	(21.5)	
≥65	18,305	(33.6)	16,647	(34.1)	1658	(29.4)	36,610	(33.6)	
Age, mean ± SD ^a^	53.8 ± 19.9						53.5 ± 19.9		0.001
Monthly income ^†^									<0.001
<15,000	20,234	(37.1)	18,153	(37.1)	2081	(36.9)	30,635	(28.1)	
15,000−19,999	25,194	(46.2)	22,584	(46.2)	2610	(46.3)	48,638	(44.6)	
≥20,000	9092	(16.7)	8142	(16.7)	950	(16.8)	29,767	(27.3)	
Urbanization level ^‡^									<0.001
1 (highest)	11,926	(21.9)	10,771	(22.0)	1155	(20.5)	30,949	(28.4)	
2	14,926	(27.4)	13,384	(27.4)	1562	(27.7)	32,473	(29.8)	
3	9185	(16.9)	8341	(17.1)	844	(15.0)	18,783	(17.2)	
4 (lowest)	18,463	(33.9)	16,383	(33.5)	2080	(36.9)	26,835	(24.6)	
Occupation category ^&^									<0.001
Office worker	22,331	(41.0)	20,110	(41.1)	2221	(39.4)	56,013	(51.4)	
Laborer	22,626	(41.5)	20,356	(41.7)	2270	(40.2)	40,255	(36.9)	
Other	9563	(17.5)	8413	(17.2)	1150	(20.4)	12,772	(11.7)	
Comorbidity									
Schizophrenia	2248	(4.12)	1797	(3.68)	451	(8.00)	426	(0.39)	<0.001
Alcohol-related illness	5223	(9.58)	3965	(8.11)	1258	(22.3)	546	(0.50)	<0.001
Anxiety	1295	(2.38)	802	(1.64)	493	(8.74)	317	(0.29)	<0.001
Mental disorders	11,425	(21.0)	11,425	(23.4)	-	-	1607	(1.47)	<0.001
Insomnia	3084	(5.66)	2055	(4.20)	1029	(18.2)	903	(0.83)	<0.001

Chi-square test; ^a^ Two sample *t*-test, total epilepsy cohort vs. comparison cohort; ^†^ New Taiwan Dollar (NTD), 1 NTD is equal to 0.03 USD; ^‡^ the urbanization level was divided by the population density of the residential area into four levels; levels 1 and 4 represent the most and least urbanized areas; ^&^ other occupation categories included individuals who were primarily retired, were unemployed, and had low income.

**Table 2:** Association of incidence and risk of SA with various risk factors.

Variable	Event	PY	Rate ^#^	Crude HR (95% CI)	Adjusted HR ^$^ (95% CI)
Epilepsy					
None	74	505,740	1.46	1.00	1.00
All	216	205,773	10.5	7.07(5.43, 9.21) ***	5.49(4.13, 7.29) ***
Epilepsy without depression	116	182,439	6.36	4.27(3.19, 5.71) ***	3.48(2.54, 4.77) ***
Epilepsy with depression	100	23,334	42.9	29.2(21.6, 39.4) ***	21.3(15.2, 30.0) ***
Age group, years					
≤49	179	361,400	4.95	1.67(1.25, 2.24) ***	1.35(1.00, 1.83)
50–64	50	154,221	3.24	1.07(0.74, 1.56)	1.01(0.69, 1.48)
≥65	61	195,892	3.11	1.00	1.00
Sex					
Women	115	267,048	4.31	1.10(0.87, 1.39)	
Men	175	444,465	3.94	1.00	1.00
Monthly income ^†^					
<15,000	104	208,409	4.99	3.87(2.47, 6.08) ***	2.46(1.51, 4.03) ***
15,000−19,999	163	321,844	5.06	3.96(2.56, 6.13) ***	2.97(1.88, 4.71) ***
≥20,000	23	181,261	1.27	1.00	1.00
Urbanization level ^‡^					
1 (highest)	53	190,218	2.79	1.00	1.00
2	78	208,561	3.74	1.34(0.95, 1.90)	1.14(0.80, 1.62)
3	50	121,394	4.12	1.48(1.00, 2.17) *	1.20(0.81, 1.77)
4 (lowest)	109	191,340	5.70	2.04(1.47, 2.83) ***	1.29(0.92, 1.81)
Occupation category ^&^					
Office worker	104	348,686	2.98	1.00	1.00
Laborer	128	270,200	4.74	1.58(1.22, 2.05) ***	1.05(0.79, 1.40)
Other	58	92,627	6.26	2.09(1.51, 2.88) ***	1.34(0.92, 1.95)
Comorbidity					
Schizophrenia					
No	274	699,592	3.92	1.00	1.00
Yes	16	11,921	13.4	3.42(2.06, 5.67) ***	1.03(0.61, 1.74)
Alcohol-related illness					
No	235	689,958	3.41	1.00	1.00
Yes	55	21,556	25.5	7.35(5.48, 9.86) ***	3.43(2.46, 4.80) ***
Anxiety					
No	280	705,118	3.97	1.00	1.00
Yes	10	6396	15.6	3.89(2.07, 7.31) ***	1.20(0.62, 2.31)
Mental disorders					
No	257	667,010	3.85	1.00	1.00
Yes	33	44,503	7.42	1.87(1.30, 2.68) ***	0.48(0.33, 0.72) ***
Insomnia					
No	265	697,831	3.80	1.00	1.00
Yes	25	13,683	18.3	4.67(3.10, 7.03) ***	1.75(1.13, 2.69) *

CI, confidence interval; HR, hazard ratio; PY, person-years; ^#^ incidence rate per 10,000 person-years; SA, suicide attempt ^$^ multivariate analysis included age, monthly income, urbanization level, and comorbidity of schizophrenia, depression, alcohol-related illness, anxiety, mental disorders, and insomnia; ^†^ New Taiwan Dollar (NTD), 1 NTD is equal to 0.03 USD; ^‡^ urbanization level was divided by the population density of the residential area into 4 levels, where level 1 represented the most urbanized areas and level 4 represented least urbanized areas; ^&^ other occupation categories included those who were primarily retired, were unemployed, and had low income * *p* < 0.05, *** *p* < 0.001.

**Table 3:** Incidence and hazard ratios for SA stratified by age, sex, income, urbanization, occupation, and comorbidity.

Variable	Comparison Cohort N = 109,040		Total Epilepsy N = 54,520			Epilepsy without Depression N = 48,879			Epilepsy with Depression N = 5641		
	Event	Rate ^#^	Event	Rate ^#^	Adjusted HR ^$^ (95% CI)	Event	Rate ^#^	Adjusted HR ^$^ (95% CI)	Event	Rate ^#^	Adjusted HR ^$^ (95% CI)
Age, year											
≤49	29	1.18	150	13.1	8.96(5.89, 13.6) ***	74	7.31	5.37(3.42, 8.45) ***	76	55.9	36.7(22.8, 59.0) ***
50–64	7	0.63	43	9.80	10.2(4.39, 23.5) ***	24	6.15	6.32(2.57, 15.5) ***	19	39.2	38.9(15.3, 99.3) ***
≥65	38	2.55	23	4.89	1.67(0.95, 2.94)	18	4.27	1.40(0.76, 2.60)	5	10.2	3.81(1.42, 10.2) **
*p* for interaction					<0.001			<0.001			
Sex											
Female	22	1.16	93	11.9	8.49(5.23, 13.8) ***	39	5.73	4.53(2.64, 7.77) ***	54	55.0	35.5(20.8, 60.5) ***
Male	52	1.64	123	9.62	4.15(2.90, 5.93) ***	77	6.73	2.93(1.98, 4.33) ***	46	34.0	13.8(8.64, 22.1) ***
*p* for interaction					0.05			0.004			
Monthly income ^†^											
<15,000	29	2.16	75	10.1	4.35(2.76, 6.87) ***	42	6.36	2.98(1.81, 4.91) ***	33	39.6	15.9(9.06, 27.8) ***
15,000−19,999	39	1.72	124	13.0	5.67(3.86, 8.33) ***	67	7.94	3.42(2.22, 5.26) ***	57	52.2	23.2(14.7, 36.6) ***
≥20,000	6	0.41	17	4.71	7.70(2.80, 21.2) ***	7	2.19	4.80(1.54, 15.0) ***	10	24.4	30.5(9.15, 101.6) ***
*p* for interaction					0.02			0.02			
Urbanization level ^‡^											
1 (highest)	9	0.62	44	9.76	12.0(5.70, 25.4) ***	23	5.72	7.29(3.24, 16.4) ***	21	42.9	51.4(21.9, 120.7) ***
2	21	1.39	57	9.94	5.70(3.34, 9.72) ***	29	5.71	3.22(1.75, 5.93) ***	28	42.8	25.6(13.7, 48.0) ***
3	16	1.85	34	9.69	3.64(1.90, 7.00) ***	16	5.07	1.81(0.82, 4.00)	18	50.6	19.9(9.12, 43.3) ***
4 (lowest)	28	2.27	81	11.9	4.33(2.72, 6.89) ***	48	8.01	3.26(1.98, 5.36) ***	33	39.6	12.2(6.79, 21.9) ***
*p* for interaction					0.01			0.004			
Occupation category ^&^											
Office worker	28	1.07	76	8.81	5.46(3.41, 8.74) ***	36	4.69	3.07(1.78, 5.28) ***	40	42.3	24.5(14.0, 42.7) ***
Laborer	31	1.66	97	11.6	5.47(3.55, 8.43) ***	58	7.80	3.80(2.37, 6.08) ***	39	42.1	18.4(10.8, 31.3) ***
Other	15	2.64	43	12.0	4.89(2.62, 9.10) ***	22	7.03	3.04(1.53, 6.04) ***	21	45.7	19.2(9.29, 39.6) ***
*p* for interaction					0.12			0.05			
Comorbidity											
None	69	1.40	120	8.64	5.25(3.89, 7.09) ***	70	5.55	3.41(2.43, 4.77) ***	50	39.0	24.7(17.1, 35.7) ***
With any one	5	4.30	96	14.4	2.76(1.11, 6.87) *	46	8.17	1.61(0.63, 4.11)	50	47.6	9.14(3.58, 23.3) ***
*p* for interaction					0.22			0.87			

CI, confidence interval; HR, hazard ratio; PY, person-years; ^#^ Incidence rate per 10,000 person-years; ^$^ multivariate analysis included age, monthly income, urbanization level, and comorbidity of schizophrenia, alcohol-related illness, anxiety, mental disorders, and insomnia; ^†^ New Taiwan Dollar (NTD), 1 NTD is equal to 0.03 USD; ^‡^ urbanization level was divided by the population density of the residential area into four levels, where level 1 represented the most urbanized areas and level 4 represented the least urbanized areas; ^&^ other occupation categories included those who were primarily retired, were unemployed, and had low income; ^§^individuals with schizophrenia, depression, alcohol-related illness, anxiety, mental disorders, and insomnia were classified into the comorbidity group; * *p* < 0.05, ** *p* < 0.01, *** *p* < 0.001.

**Table 4:** Comparison of hazard ratios for suicide attempt stratified by age, sex, and comorbidities in patients with epilepsy with and without comorbid depression.

Variable	Epilepsy without Depression (N = 48,879)	Epilepsy with Depression (N = 5641)
	Adjusted HR ^$^ (95% CI)	Adjusted HR ^$^ (95% CI)
All	1.00	6.03(4.40, 8.28) ***
Age, year		
≤49	1.00	6.75(4.60, 9.91) ***
50–64	1.00	5.69(2.80, 11.6) ***
≥65	1.00	2.58(0.88, 7.52)
*p* for interaction		0.03
Sex		
Female	1.00	7.98(5.05, 12.6) ***
Male	1.00	4.49(2.86, 7.04) ***
*p* for interaction		0.02
Monthly income ^†^		
<15,000	1.00	5.38(3.20, 9.05) ***
15,000−19,999	1.00	6.53(4.25, 10.0) ***
≥20,000	1.00	6.38(2.03, 20.0) **
*p* for interaction		0.48
Urbanization level ^‡^		
1 (highest)	1.00	7.43(3.66, 15.1) ***
2	1.00	7.53(4.13, 13.7) ***
3	1.00	11.8(4.97, 27.9) ***
4 (lowest)	1.00	3.55(2.10, 5.99) ***
*p* for interaction		0.22
Occupation category ^&^		
Office worker	1.00	7.75(4.48, 13.4) ***
Laborer	1.00	4.77(2.93, 7.75) ***
Other	1.00	6.16(3.20, 11.8) ***
*p* for interaction		0.25
Comorbidity ^§^		
None	1.00	7.50(5.20, 10.8) ***
With any one	1.00	5.64(3.77, 8.44) ***
*p* for interaction		0.52

CI, confidence interval; HR, hazard ratio; ^$^ multivariate analysis included age, monthly income, urbanization level, and comorbidity of schizophrenia, alcohol-related illness, anxiety, mental disorders, and insomnia; ^†^ New Taiwan Dollar (NTD), 1 NTD is equal to 0.03 USD; ^‡^ urbanization level was divided by the population density of the residential area into four levels, where level 1 represented the most urbanized areas and level 4 represented the least urbanized areas; ^&^ other occupation categories included those who were primarily retired, were unemployed, and had low income; ^§^ individuals with schizophrenia, depression, alcohol-related illness, anxiety, mental disorders, and insomnia were classified into the comorbidity group; ** *p* < 0.01, *** *p* < 0.001.

**Table 5:** Incidence and hazard ratios of suicidal drug overdose in patients with epilepsy stratified according to the presence or absence of comorbid depression by Cox method.

Variable	Comparison Cohort	Total Epilepsy	Epilepsy without Depression	Epilepsy with Depression
	(N = 109,040)	(N = 54,520)	(N = 48,879)	(N = 5641)
Person-years	505,427	203,448	180,547	39.3
Event, *n*	196	932	709	223
Rate ^#^	3.88	45.8	39.3	97.4
Crude HR (95% CI)	1(Reference)	11.5(9.86, 13.4) ***	9.84(8.40, 11.5) ***	24.8(20.5, 30.1) ***
Adjusted HR ^$^ (95% CI)	1(Reference)	10.6(8.99, 12.4) ***	9.01(7.63, 10.6) ***	22.9(18.7, 28.2) ***
Crude HR (95% CI)			1(Reference)	2.53(2.18, 2.94) ***
Adjusted HR ^$^ (95% CI)			1(Reference)	2.56(2.17, 3.04) ***

CI, confidence interval; HR, hazard ratio; ^#^ incidence rate per 10,000 person-years; ^$^ multivariate analysis included age, monthly income, urbanization level, and comorbidity of schizophrenia, alcohol-related illness, anxiety, mental disorders, and insomnia; *** *p* < 0.001.

## Abbreviations

aHR	adjusted hazard ratio
CI	confidence interval
NHIRD	National Health Insurance Research Database
ICD-9-CM	International Classification of Diseases, Ninth Revision, Clinical Modification
